# Structural connectome alterations in anxious dogs: a DTI-based study

**DOI:** 10.1038/s41598-023-37121-0

**Published:** 2023-06-19

**Authors:** Qinyuan Chen, Yangfeng Xu, Emma Christiaen, Guo-Rong Wu, Sara De Witte, Christian Vanhove, Jimmy Saunders, Kathelijne Peremans, Chris Baeken

**Affiliations:** 1 Ghent Experimental Psychiatry (GHEP) Lab, Department of Head and Skin, Faculty of Medicine and Health Sciences, Ghent University, Ghent, Belgium; 2 Department of Morphology, Imaging, Orthopedics, Rehabilitation and Nutrition, Faculty of Veterinary Medicine, Ghent University, Merelbeke, Belgium; 3 Medical Image and Signal Processing (MEDISIP), Department of Electronics and Information Systems, Faculty of Engineering and Architecture, Ghent University, Ghent, Belgium; 4 Key Laboratory of Cognition and Personality, Faculty of Psychology, Southwest University, Chongqing, China; 5 School of Psychology, Jiangxi Normal University, Nanchang, China; 6 Department of Neurology and Bru-BRAIN, University Hospital (UZ Brussel), Brussels, Belgium; 7 Neuroprotection & Neuromodulation Research Group (NEUR), Center for Neurosciences (C4N), Vrije Universiteit Brussel (VUB), Brussels, Belgium; 8 Vrije Universiteit Brussel (VUB), Department of Psychiatry, University Hospital (UZ Brussel), Brussels, Belgium; 9 Department of Electrical Engineering, Eindhoven University of Technology, Eindhoven, The Netherlands

**Keywords:** Anxiety, Neuroscience, Biomarkers

## Abstract

Anxiety and fear are dysfunctional behaviors commonly observed in domesticated dogs. Although dogs and humans share psychopathological similarities, little is known about how dysfunctional fear behaviors are represented in brain networks in dogs diagnosed with anxiety disorders. A combination of diffusion tensor imaging (DTI) and graph theory was used to investigate the underlying structural connections of dysfunctional anxiety in anxious dogs and compared with healthy dogs with normal behavior. The degree of anxiety was assessed using the Canine Behavioral Assessment & Research Questionnaire (C-BARQ), a widely used, validated questionnaire for abnormal behaviors in dogs. Anxious dogs showed significantly decreased clustering coefficient (${C}_{p}$), decreased global efficiency (${E}_{glob}$), and increased small-worldness (σ) when compared with healthy dogs. The nodal parameters that differed between the anxious dogs and healthy dogs were mainly located in the posterior part of the brain, including the occipital lobe, posterior cingulate gyrus, hippocampus, mesencephalon, and cerebellum. Furthermore, the nodal degree (${K}_{i}$) of the left cerebellum was significantly negatively correlated with “excitability” in the C-BARQ of anxious dogs. These findings could contribute to the understanding of a disrupted brain structural connectome underlying the pathological mechanisms of anxiety-related disorders in dogs.

Fear and anxiety are commonly observed behaviors that manifest across various species of mammals and beyond, which becomes most evident when confronted with danger. The behavioral characteristics in humans and animals share excessive fear, anxiety, but also related anxiety disorders^1,2^. It is also important to note that the dog, man's best friend, shares many psychopathological behaviors with human, including similar neurobiological patterns when exhibiting anxiety^3,4^. As in humans, the prevalence of anxiety disorders in dogs is high, and it is the most common behavioral disorder in daily veterinary practice^5^. It can be confidently stated that anxiety in dogs is a serious welfare problem, not only for the welfare of the animal, but it may also jeopardize the relationship with the owner, leading to abandonment, rehoming or even euthanasia^6^. In the case of comorbid aggression, anxious behavior can pose safety risks, not only to other animals but also to humans. Given the close relationship between these two species, understanding more about the anxious dog can help to select the adequate treatment, such as behavioral therapy and pharmacotherapy.

However, a better understanding of brain functioning in dogs will allow us to provide better interventions. Although specific brain regions correlated with anxiety have been reported in primates^7,8^, rodents^9,10^, and dogs with pathological anxiety^11^, it remains to be seen whether pathological anxiety shows similar structural influences on neuronal function in dogs and humans. Diffusion tensor imaging (DTI) is an non-invasive technique that measures the diffusion of water molecules in tissues, and provides useful information about microstructure of brain white matter (WM) in vivo^12^. It has been widely used for studying brain structural connectivity in human populations, as well as in psychiatric and neurological diseases that are characterized by WM deficits, such as depression^13^, anxiety disorders^14^, and Alzheimer’s disease^15^. Furthermore, graph theoretical approaches have been widely applied to connectome studies to investigate the topological organization alterations in neuropsychiatric disorders (e.g. anxiety disorders), enabling understanding of how brain disorders affect cognition and behaviors based on fundamental properties of the brain network^16,17^.

To close this gap in knowledge, in this structural connectome study, a combination of DTI and graph theory was used to investigate the underlying structural neuronal connections of pathological anxiety in dogs. We obtained DTI data from dogs diagnosed with anxiety disorder and healthy dogs (without any abnormal behavior). In addition, various symptoms of anxiety were assessed using the Canine Behavioral Assessment & Research Questionnaire (C-BARQ), a common behavioral abnormality questionnaire for dogs. The aim of this study was twofold: (1) to evaluate differences in topology of structural brain networks between healthy dogs and dogs with anxiety disorders; (2) to assess whether different symptoms of anxiety (C-BARQ) are related to specific structural network differences in the anxious group.

## Materials and methods

### Animals

Eleven dogs (see Supplementary Table S1) were recruited as the patient group. All dogs in the patient group were diagnosed with anxious behavior (with or without aggression) by veterinarians based on the dog’s history, physical examination, and questionnaires. Blood samples were taken for thyroid function tests, including thyroid stimulating hormone (TSH) and thyroxine (T4), to exclude thyroid dysfunction-led behavior problems in anxious dogs^18^. The owners of the anxious dogs were informed that they were participating in a study about anxiety and clinical disease status in dogs and informed consent was provided. In addition, fifteen beagle dogs (3 castrated males and 12 castrated females; aged between 12 and 96 months) were recruited as the healthy group. These dogs were owned by the Department of Small Animals, and the Department of Veterinary Medical Imaging and Small Animal Orthopedics, Ghent University, Belgium. Following the federal welfare rules, all healthy dogs were housed in groups of eight on a 15 m^2^ internal surface, with access to an outside area of 15 m^2^. The floor covering in the inner part consisted of wood shavings. Toys were given to these dogs every day and they were released to an enclosed playground. In addition, these dogs lived in an environment handled by the same caretakers, including daily walking, free open space running, and regular time of feeding. Furthermore, these dogs were evaluated regularly by both veterinarians and caretakers using a local monitoring protocol by Ghent University Ethical Committee (Monitoring of welfare in dogs kept and used for research purposes by the Ghent University Ethical Committee). None of them displayed anxiety or any other deviant behavior during the whole study. This study was approved by the local Ethical Committee of Ghent University and the Belgian Deontological Committee (reference number: 2015-140, 2018-09, 2018-088). All procedures were performed in accordance with relevant guidelines and regulations. The reporting in the manuscript follows the recommendations in the ARRIVE guidelines^19^.

### Behavioral assessment

The frequency and severity of problematic behaviors in anxious dogs were assessed using the Canine Behavioral Assessment & Research Questionnaire (C-BARQ)^20,21^. This questionnaire, consisting of 101 items, is a validated and standardized evaluation tool for measuring both behavior and behavior problems in dogs^22^. The C-BARQ divides behavior into seven sections, including “excitability”, “aggression”, “fear and anxiety”, “attachment and attention seeking”, “separation-related behaviors”, “obedience”, and “miscellaneous”. All C-BARQ items were evaluated using a five-point scale and scored by the owners. Supplementary Table S2 lists the C-BARQ scores for all anxious dogs. A questionnaire designed for the current research was used to get an overview of the different anxious and aggression-driven reactions in different situations, including the owner’s information in addition to their dog’s history, including separation anxiety and noise phobia/reactivity screen, reactivity and aggression screen, and repetitive and ritual behavior.

### MRI data acquisition

All imaging data were acquired on a 3 T Siemens MAGNETOM Prisma scanner with a phased-array spine coil and a phased-array body matrix coil at the University Hospital Ghent (UZ Gent), Ghent University, Belgium. Diffusion MRI (dMRI) data were obtained using a twice-reinforced spin-echo diffusion-weighted echo-planar imaging (EPI) sequence with the following parameters: repetition time (TR) = 7800 ms, echo time (TE) = 120 ms, field of view (FOV) = 220 mm × 220 mm, voxel size = 2 × 2 × 2.2 mm^3^, slice thickness = 2 mm, one volume with *b* = 0 s/mm^2^, 64 volumes with *b* = 2800 s/mm^2^, and 50 contiguous slices. High resolution brain structural images were obtained using a T1-weighted 3D Magnetization-Prepared Rapid Gradient Echo (MP-RAGE) sequence (TR = 2250 ms, TE = 4.18 ms, flip angle = 9°, slice thickness = 1 mm, data matrix = 256 × 256, FOV = 256 mm × 256 mm, and voxel size = 1 × 1 × 1 mm^3^, and 176 slices).

Before the MRI scan procedure, all dogs were pre-medicated in a quiet room with dexmedetomidine (Dexdomitor; Orion) at 375 μg/m^2^ by intramuscular injection. General anesthesia was induced with 2–3 mg/kg propofol (Propovet Multidose, Abbott Laboratories) intravenously through a cephalic vein catheter. Anesthesia was performed by a veterinary (Y.X), and maintained with isoflurane (Isoflo, Abbott Laboratories) in oxygen given to effect.

### Data preprocessing

The dMRI data were preprocessed using MRtrix3 (http://www.brain.org.au/software/)^23^ and ExploreDTI (version 4.8.6, https://www.exploredti.com/). First, denoising^24^ and cropping were performed in MRtirx3. Subsequently, the dMRI data were corrected for signal drift, Gibbs ringing artefacts^25^, motion, eddy current distortions and susceptibility induced distortions in ExploreDTI. Quality assessment was performed for the corrected data by checking the average residuals across the DWIs for each voxel used the data quality summary in ExploreDTI. In MRtrix3, single-shell response functions were estimated using the iterative algorithm proposed in Tournier et al.^26^. The pre-processed dMRI data were then upsampled to a voxel size of 1.25 mm isotropic to improve anatomical details. The brain mask was drawn manually for each canine using MRView (the MRtrix image viewer). Then, the fiber orientation distributions (FODs) were estimated using single-shell constrained spherical deconvolution (CSD)^27^. Finally, the resulting FOD image from each dog were registered to a study-specific unbiased FOD template with reorientation^28^. This template was generated for all 26 dogs using linear and nonlinear registration of these canine FOD images^29^. We also create a study-specific T1-weighted template for all dogs.

### Structural connectome construction

The canine brain structural connectome was constructed based on a parcellated atlas used in our previous study^30^, which parcellates the canine brain into 30 cortical and subcortical regions of interest (ROIs). The atlas was adapted based on former research^31,32^, and constructed based on T1-weighted anatomical images of all canines. For a list of these ROIs, see Supplementary Table S3.

**Figure 1:**
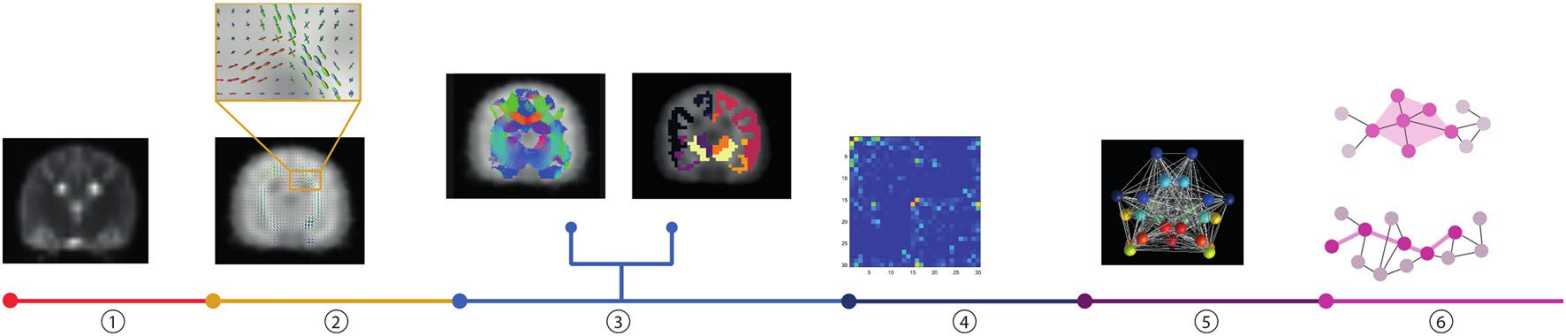
A flowchart for the construction of the structural connectome in canine brain. (1) diffusion weighted image preprocessing; (2) fiber orientation distributions (FODs) estimation; (3) the segmented 30 ROIs in the FOD template space (picture in the right side) and a whole brain probabilistic tractography approach was used to reconstruct the white matter pathway (picture in the left side); (4) the fiber density was taken to represent the weight of the connection and was aggregated into a structural connectivity matrix; (5) a structural connectome or graph, in which the nodes correspond with the ROIs and the edges is constructed based on the structural connectivity matrix; and (6) global and nodal network metrics were calculated to quantify the network.

For each canine, we defined each ROI as a node and the number of streamlines that corss each pair of ROIs as edges. We first co-registered the T1-weighted template to the FOD template using nonlinear transformations. Inverse transformations were then used to warped the atlas from T1-weighted template space to FOD template space using a nearest-neighbor interpolation method. From this, we obtained the segmented 30 ROIs in FOD template space. Whole-brain probabilistic tractography was performed using 2nd order integration over fiber orientation distributions (iFOD2) algorithm^33^. Ten million streamlines were generated by randomly putting seeds in WM within the FOD template. Tracking parameters were included a step size of 0.1 mm, FOD cutoff threshold of 0.2, and maximum angle of 45 degrees. Next, Spherical-deconvolution informed filtering of tractograms (SIFT) algorithm^34^ was applied to obtain a better match between the reconstructed streamlines and the underlying WM structures, provided a highly biologically relevant measure of ‘structural connectivity’^35^. The edge weight was determined by the number of streamlines (NOS). In this way, we obtained a 30 × 30 NOS-weighted connectivity matrix that represent the whole-brain structural connectome for each canine. Figure 1 depicts the main steps for constructing the structural connectome.

### Graph analysis

Graph theoretical network metrics were computed to examine the possible differences in topological properties of the whole-brain WM structural connectome between different groups using GRETNA toolbox (https://www.nitrc.org/projects/gretna)^36^ in MATLAB. We characterized the global properties of the canine brain networks by the following five parameters: clustering coefficient (${C}_{p}$), global efficiency (${E}_{glob}$), local efficiency (${E}_{loc}$), shortest path length (${L}_{p}$), and small-worldness (σ). Specifically, ${C}_{p}$ measures the degree of nodes tending to cluster together, ${E}_{glob}$ measures the efficiency of parallel information transfer through the network, ${E}_{loc}$ measures the efficiency of information exchange within a local subnetwork or among adjacent regions, ${L}_{p}$ measures the ability for information propagation within the network, the small-worldness indicates a typical network that has similar path length but higher clustering than a random network. In addition, three nodal parameters, including nodal degree (${K}_{i}$), nodal efficiency (${E}_{i}$), and betweenness centrality (BC) were calculated and used to characterize the nodal properties of the canine brain networks. The definitions and interpretations of these topological parameters are listed in Supplementary Table S4 or can be found in previous studies^37,38^.

The connectivity matrix was thresholded by sparsity values ranging from 20 to 40% with a 5% interval to ensure that the number of nodes and connections were matched across dogs and groups^39^. The sparsity of a network was defined as the existing number of edges in the network divided by the maximum possible number of the edges^40^. The network metrics were calculated at each threshold value and averaged over these values.

### Statistical analysis

#### Comparison of animal characteristics

An independent *t*-test was used to determine the group difference in age. A *χ*^*2*^-test was applied to determine the group difference in gender. These statistical analyses were performed using the Statistical Package for the Social Science (SPSS) version 28 (IBM, Chicago). The significance level was set at *p* < 0.05, two-tailed, for all analyses.

#### Comparison of network parameters

The between-group differences in network parameters (global and nodal properties) were determined using a non-parametric permutation test. This permutation procedure was repeated 10,000 times to obtain an empirical distribution of the difference. To correct the network parameters for multiple comparisons, we applied FDR correction by using *p* < 0.05 as the significant threshold. We took age and gender as covariates and regressed them out when comparing between-group differences in network parameters.

To further explore the correlations between abnormal network properties and the severity of anxious symptoms, Spearman’s partial correlation coefficient was performed in the patient group. We first extracted the network parameters with significantly between-group differences for each dog of the patient group, then calculated Spearman’s partial correlation between each of these parameters and the scores of C-BARQ. We controlled age as covariates and applied false discovery rate (FDR) correction for multiple comparisons. The threshold for significant level was set at *p* < 0.05.

## Results

### Demographic and clinical information

Table 1 lists the demographic data for all dogs. No significant difference between the two groups were found for gender. An independent *t*-test showed significant differences in age between the two groups (*p* < 0.001).

**Table 1:** Demographic information of anxious dogs and healthy beagles.

Characteristic	Anxious group	Healthy group	*p* value
	(*n* = 11)	(*n* = 15)	
Age (months, mean ± SD)	81.55 ± 30.61	42.4 ± 19.7	< 0.001
Gender (FC/MC)	5/6	12/3	0.103

An independent *t*-test was used to detect between-group differences in age. A *χ*^*2*^-test was used to detect differences in gender between the two groups (*p* < 0.05, two-sided).

*SD* Standard deviation, *FC* Castrated female, *MC* Castrated male.

### Global topological properties of structural connectome

**Figure 2:**
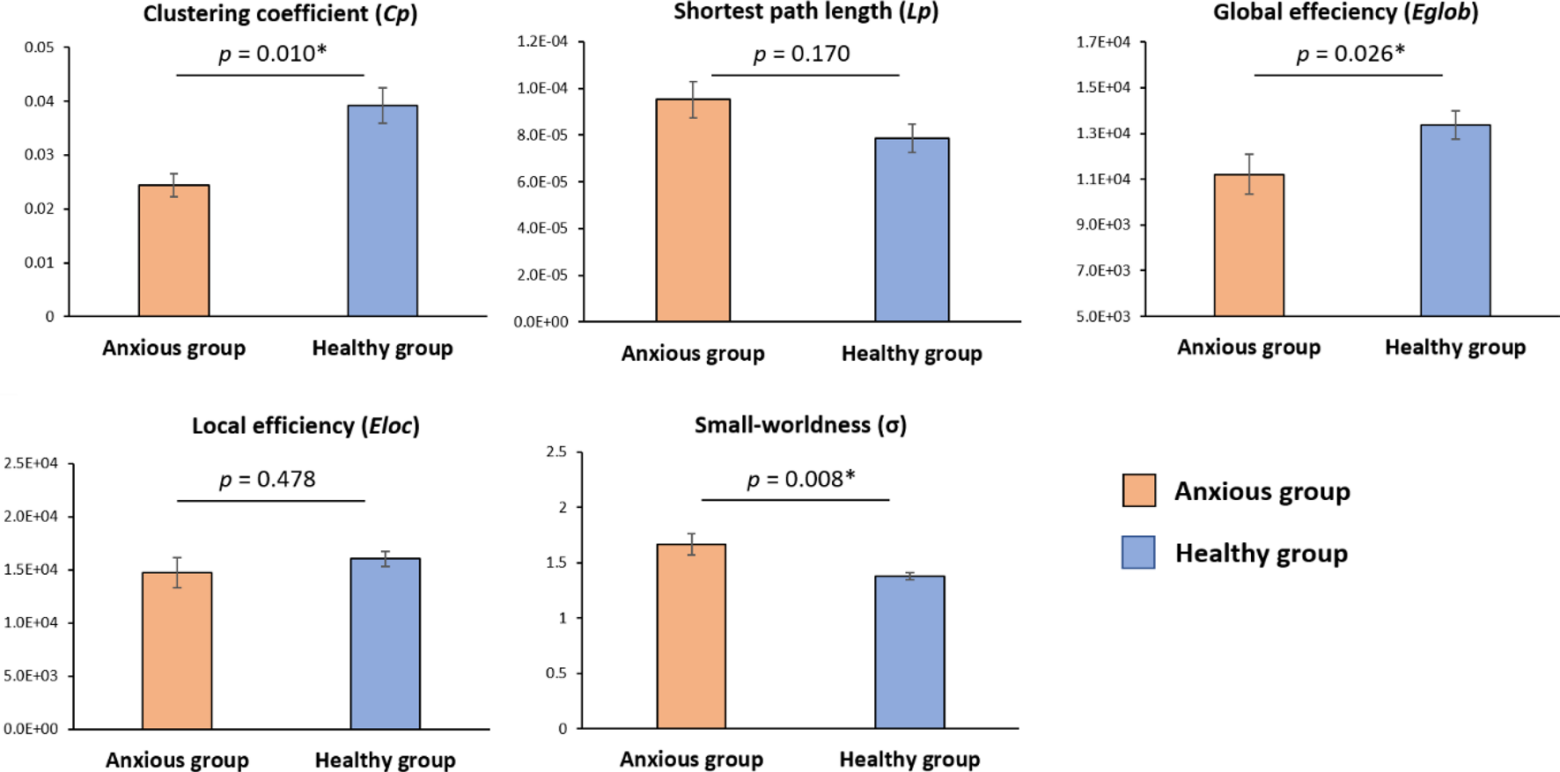
Global parameters of the canine structural brain networks of the anxious and healthy groups. The σ > 1 for both anxiety and healthy groups, indicating that the canine brain is a small-world network. The anxiety group showed significantly decreased clustering coefficient and global efficiency compared with the healthy group (10,000 permutation, FDR correction). The asterisk symbol indicates significant between-group difference in the given global parameter at the *p* < 0.05 level after FDR correction.

Figure 2 shows the global parameters of the canine structural brain networks of the anxious and the healthy groups. We found σ > 1 for the two groups, indicating that both anxious and healthy groups exhibited small-world attributes. The anxious group showed significantly lower ${C}_{p}$ (*p* = 0.01), and higher σ (*p* = 0.0076) compared with the healthy group (10,000 permutation, FDR correction). No significant differences were found in other global topological parameters.

### Nodal topological properties of structural connectome

**Figure 3:**
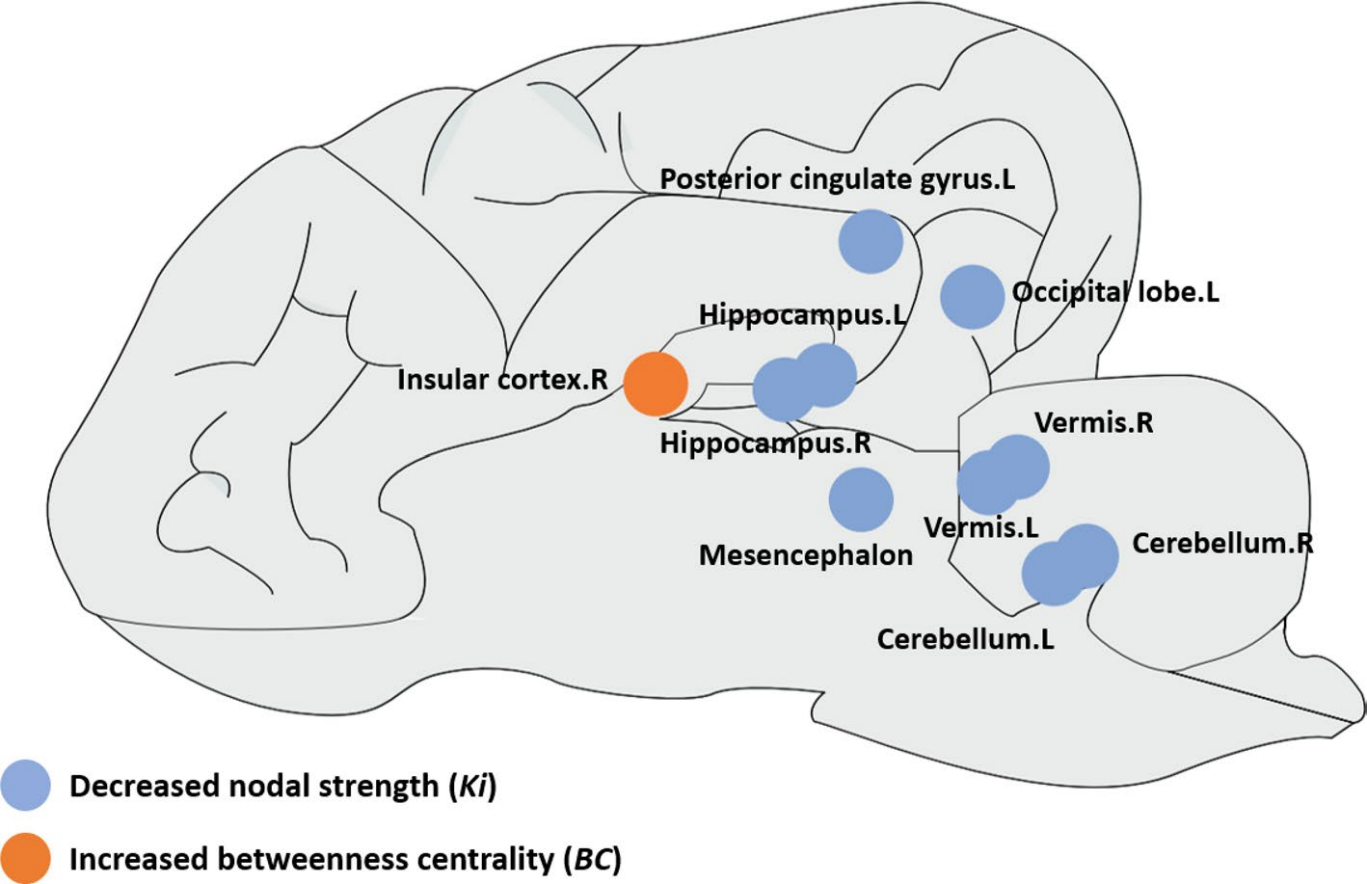
Brain regions with significant between-group differences in the nodal parameters of the canine structural brain networks between the anxious and healthy groups. Details of the nodal parameters for these brain regions are listed in Table 2. *L (R)* left (right) hemisphere.

Figure 3 and Table 2 shows the between-group differences in the nodal parameters of the canine structural brain networks. The anxious group exhibited significantly decreased ${K}_{i}$ in the left occipital lobe (*p* = 0.007), left posterior cingulate gyrus (*p* = 0.013), bilateral hippocampus (the left hippocampus, *p* = 0.003; the right hippocampus, *p* = 0.015), cerebellum (the left cerebellum, *p* = 0.003; the right cerebellum, *p* = 0.001), bilateral vermis (the left vermis, *p* = 0.008; the right vermis, *p* = 0.003), and mesencephalon (*p* = 0.0004) compared with the healthy group (10,000 permutation, FDR correction). We also found that the anxious group showed significantly increased BC in the right insular cortex (*p* = 0.0001) compared with the healthy group (10,000 permutation, FDR correction).

**Table 2:** Brain regions showing significant differences in nodal parameters (nodal degree *Ki*, betweenness centrality *BC*) of canine structural brain networks between the anxious and healthy groups.

Parameters	Brain regions	Anxious group		Healthy group		*p* value
		Mean	SD	Mean	SD	
*Ki*	Occipital. L	1.25E+05	8.59E+04	2.53E+05	7.27E+04	0.0070
	Posterior cingulate gyrus. L	1.11E+05	4.87E+04	1.76E+05	5.19E+04	0.0130
	Hippocampus. L	5.60E+04	2.88E+04	1.14E+05	3.93E+04	0.0030
	Hippocampus. R	8.20E+04	3.92E+04	1.34E+05	3.16E+04	0.0150
	Mesencephalon	1.34E+05	6.36E+04	2.72E+05	5.81E+04	0.0004
	Vermis. L	4.46E+04	2.86E+04	8.53E+04	2.64E+04	0.0080
	Vermis. R	5.61E+04	4.38E+04	1.33E+05	4.65E+04	0.0030
	Cerebellum. L	5.27E+04	3.01E+04	1.30E+05	5.32E+04	0.0030
	Cerebellum. R	4.00E+04	3.14E+04	9.73E+04	3.21E+04	0.0010
*BC*	Insular cortex. R	128.30	108.30	66.3	32.2	0.0001

*L (R)* left (right) hemisphere, *SD* standard deviation.

### Relationship between network properties and clinical measures

**Figure 4:**
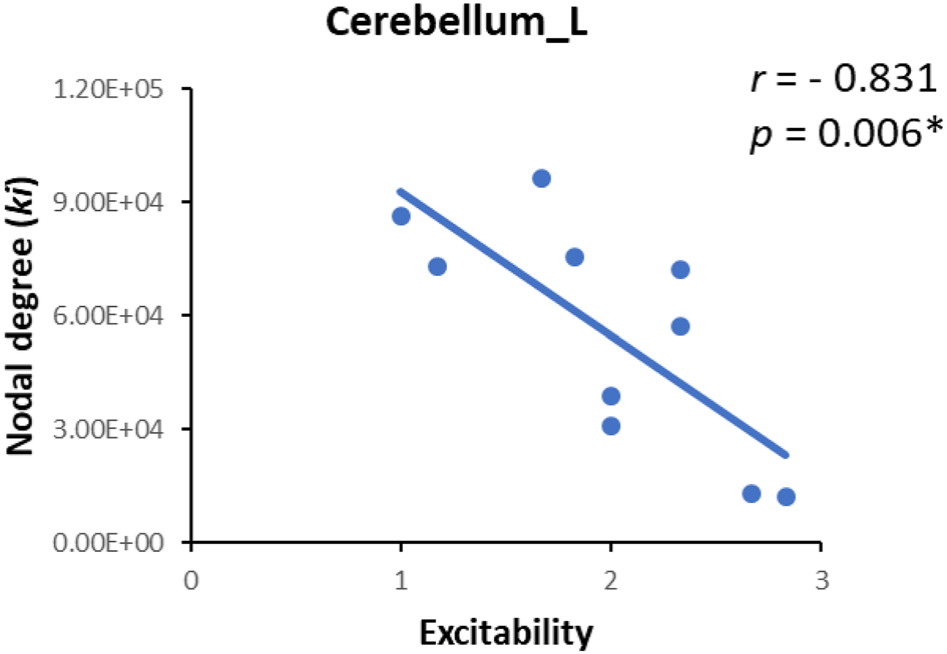
Correlations between network metrics and C-BARQ scores. Nodal degree (ki) in the left cerebellum negatively correlated with “excitability” of C-BARQ in the anxious group. Cerebellum_L, left cerebellum. The asterisk symbol indicates significant correlation between network metrics and C-BARQ scores at *p* < 0.05 level after FDR correction.

For each of the altered network parameters in the group comparison, we estimated the correlations between the altered network parameters and the C-BARQ scores in the patient group. The nodal degree in the left cerebellum was significantly negatively correlated with the scores of “excitability” section (*r* = − 0.831, *p* = 0.006, FDR corrected) in the patient group (see Fig. 4). No other global or local topological properties was found to be related with C-BARQ scores in the patient group.

## Discussion

In this structural connectome study, we evaluated the abnormal properties of the brain structural connectome in dogs diagnosed with anxiety-related disorders and compared them with healthy dogs. We found significant differences in global and nodal parameters between the anxious and non-anxious group. Specifically, decreases in segregation (${C}_{p}$) and integration (${E}_{glob}$), and an increase in small-worldness (σ) were found in the dogs displaying anxiety-related behaviors. The nodes with between-group differences in the nodal parameters (${K}_{i}$, *BC*) were mainly located in the occipital, limbic regions, mesencephalon, and the cerebellum. Furthermore, the anxious dogs showed a negative correlation between the nodal degree (${K}_{i}$) in the left cerebellum and “excitability” scores obtained with the C-BARQ, indicating that the excessive behavior in the anxious dogs was structurally disconnected between the left cerebellum and the rest of the brain regions within the network.

The brain structural connectome of both anxious and healthy dogs showed small-world topology (σ > 1), suggesting that the dog brain characterizes a small-world network which allows high efficiency of information flow at low wiring costs for both local and long distance within the brain network. Our finding of small-world networks in the canine brain is consistent with previous studies in humans and animals. For example, human studies have pointed to robust features of small-worldness in functional and structural brain networks^40,41^, and similar small-world networks were found in macaque, cat, and rodent brains^42–45^. The findings of our study confirm the efficiency of information transfer between brain regions also in the dog brain, regardless of the anxiety state.

Nevertheless, the small-worldness was significantly higher in the group of anxious dogs compared to the healthy dogs. Of interest, Yang et al.^46^ also found an increase in small-worldness associated with reduced working memory task performance in a subgroup of (human) patients displaying severe depression and anxiety symptoms. Previous studies have documented anxiety-related disorders associated with the impairment of cognitive processing in human and other animals, such as decreased decision-making abilities, and executive control (i.e., inhibition, shifting, and updating)^47–52^. In addition, higher small-world networks undergo faster signal propagation, which may result in neuropsychiatric symptoms being more easily spread within the networks^53,54^.

Furthermore, it has been documented that abnormalities in functional integration and segregation are key features of brain network disorganization in neuropsychiatric disorders^17,55^, including mood and anxiety disorders^56^, schizophrenia^57^, and Alzheimer disease^58^. Consistent with these study findings, in the present study, we observed that dogs diagnosed with anxiety-related disorders showed significantly decreased ${C}_{p}$ (segregation) and ${E}_{glob}$ (integration) than the healthy dogs. Clustering coefficient (${C}_{p}$), which is a measure of functional segregation in the brain networks, demonstrates the probability that two nodes connected to a third node are also connected to each other^38^. This interconnectivity of nodes in the brain networks can ‘entrap’ pathological processes, preventing them from spreading to other areas of the networks^59^. The significantly lower ${C}_{p}$ in anxious dogs compared to healthy dogs suggests sparse interconnected nodes in the structural connectome of anxious dogs, resulting in less resilience of the brain networks to the disease. As a measure of functional integration in the brain networks, global efficiency (${E}_{glob}$) reflects the capability of a network for parallel information transfer between nodes (i.e., brain regions) via multiple series of edges (i.e., WM tracts) in the structural connectome^40,60^. Both animal model and human studies^61–63^ proposed that anxiety-related disorders have been linked to alterations in WM tracts, which may affect the anatomical projection between brain network regions^59^. Along with this reasoning, the decrease in global efficiency in anxious dogs could be attributed to the degeneration of WM tracts for information transmission^64^. Our result of altered global network parameters is also in line with previous studies, suggesting that the altered information transfer in the structural connectome of anxious dogs relates to the emergence of anxiety-related disorders^56,64,65^.

Compared with healthy dogs, at the nodal level, we detected a significantly decreased nodal degree (${K}_{i}$) in the left occipital lobe, left posterior cingulate gyrus, bilateral hippocampus, cerebellum, bilateral vermis, and mesencephalon (see Fig. 3 and Table 2) in anxious dogs. Of interest, these brain regions have been widely reported to show functional and structural abnormalities in anxiety-related disorders^11,47,66–73^. The decreased nodal degree in these brain regions refers to a reduction in their structural connections with other brain regions, possibly resulting in a more isolated situation and less interregional information exchange. These differences in the nodal degree indicate changes in organization and connectivity, and potentially reflect issues with the information communication ability within the brain networks, which may have implications for brain function and disruptive behaviors.

As in humans and non-human primates, the occipital lobe is associated with visual information processing in dogs^74–76^. Furthermore, the occipital brain regions also play a role in the processing of emotionally-relevant visual information, such as stimuli associated with threat or danger, which may contribute to the development or maintenance of anxiety symptoms and disorders^66,75,77^. For instance, occipital cortical 5-HT2A receptor down-regulation relating to an hyperactive serotonergic system has been found in dogs with anxiety disorders^6^. Of interest compared with healthy controls, Yang et al.^14^ found decreased nodal degree in the occipital brain areas of structural connectome in (human) patients with generalized anxiety disorder, which demonstrated stronger sensitivity and over-processing of emotional visual information. Moreover, Lai^78^ suggested that the connectivity between occipital lobe and other brain regions could be impaired due to excessive anxiety and fear, which is also consistent with our findings of decreased nodal degree in the occipital lobe in anxious dogs.

In humans, the posterior cingulate cortex has been identified as a crucial hub region of the Default Mode Network (DMN), which has been repeatedly found to be implicated in the pathophysiology of many psychiatric disorders, including anxiety disorders^47,79^. Although some previous resting-state fMRI studies in anesthetized and awake dogs identified an anterior and posterior DMN component^80,81^, no study yet has revealed how DMN plays a role in dog brain. Our current findings indicate that in anxious dogs the posterior parts of the DMN are involved in line with human psychopathological findings. In addition, it has been demonstrated that the posterior cingulate cortex is associated with retrieval of episodic memories which are stored in the hippocampus and evaluates the emotional content of relevant information from the sensory system^81–83^. Evidence from animal and human studies examining hippocampus function suggests its primary role in contextual learning, and fear modulating^84–86^. Disrupted function and structural deficits in the hippocampus are reported in both animal and human models of anxiety, pointing to a crucial role of hippocampus in fear generation and fear modulation^87,88^. In the present study, the altered nodal properties in the posterior cingulate cortex, together with the hippocampus and the occipital lobes (sensory system) may suggest that alterations in these interacted brain regions may cause dysfunctional emotional information processing in anxious dogs.

In addition, we found a decreased nodal degree in the cerebellum, bilateral vermis, and mesencephalon of anxious dogs. Notwithstanding that the cerebellum and vermis are traditionally associated with motor functions, recent studies demonstrated that the cerebellum and vermis are also involved in anxiety disorders, such as fear memory consolidation^89,90^, and prediction of future threat^91,92^. Animal and human models also proposed that the cerebellum and vermis play an important role in fear and avoidant behaviors^93–95^. Furthermore, rodent studies revealed that midbrain structures, such as the mesencephalon, implicated in the responses to the fear- or anxiety-inducing stimuli, substantiating the midbrain as an important part of underlying neural circuitries of anxiety disorders^96–98^. Lastly, we found significantly increased betweenness centrality (BC) in the right insular cortex in anxious dogs (see Fig. 3 and Table 2), compared with their healthy counterparts. It is important to note that increased betweenness centrality in brain networks refers to a higher value of betweenness centrality for a particular node in the network compared to other nodes. Here, the increased betweenness centrality for the right insula within the brain network indicates a more central and influential role for this brain area in the brain network of anxious dogs. The insular cortex has been consistently reported to be implicated in anxiety disorders across different species from rodents to humans^99,100^, supporting a role for the insular cortex in mediating fear and anxiety. For instance, functional neuroimaging studies in both animal and human models showed enhanced insula reactivity in anxiety disorders^101^. Moreover, the insula is a brain region involved in various functions such as interoception, empathy and emotional processing. Previous studies proposed that changes in insular-mediated anticipation and prediction of future events may lead to heightened anxiety^102^. The findings of our study suggested that changes in the structural network organization in the right insula may contribute to anxiety disorders in dogs.

To further investigate the relationship between altered network properties and behavior of anxious dogs, we detect the correlations between altered network parameters in between-group comparison and C-BARQ scores. The nodal degree in the left cerebellum was found to be negatively correlated with scores of "excitability" on the C-BARQ questionnaire in anxious dogs (see Fig. 4). Previous studies demonstrated that excitability indicates a high emotional arousal in response to external stimuli or events, which is associated with anxiety-related disorders^103–105^. Both in humans and dogs, high levels of arousal are associated with impairment of top-down cognitive control, which in turn has been linked to anxiety^106,107^. Specifically, the lower nodal degree in the left cerebellum of the structural connectome, the worse behaviors exhibited in dogs with anxiety disorders. This result is consistent with recent studies both in humans and animals that illustrates that the cerebellum plays a crucial role in fear and anxiety-related behaviors^91,108–110^. Structural and functional abnormalities in the cerebellum may lead to the expression of exacerbated fear and anxiety-related behaviors toward situations that do not suppose a real threat^89,111^. In addition, patients with cerebellar lesions will present anxiety-related behavior, and some were diagnosed with anxiety-related disorders^112,113^. In animal models, loss of cerebellar function manipulations resulted in impaired anxiety-related behaviors^93,109,114^. Taken together, our findings suggested the decreased nodal properties in the left cerebellum may lead to the expression of excessive anxiety behaviors, providing evidence that the left cerebellum may play an important role in anxious behaviors in dogs.

Several limitations should be taken into account. First, the sample size of this study is relatively small, due to our ethical restrictions. In addition, it should be noted that the healthy control group consisted only of healthy beagles and that the patient group consisted of different breeds (see also supplemental material) which may have introduced some biases in the analysis. This can be refined in the future when we have enough patients with the same breed as the control group, then we can make a more direct comparison. Second, the different housing environment between the two groups should be also considered. With the current data, we are not able to disentangle the effect of housing environment on the anxiety symptoms in this cohort. The future studies could use a more naturalistic healthy canine as the control group. Moreover, we used C-BARQ to conduct behavioral assessment for the anxious group and used the welfare dog monitoring protocol by the Ghent University Ethical Committee to conduct behavioral assessment regularly for the healthy group, which limits the investigation of the correlations between the abnormal brain regions and behaviors across two groups. Finally, the frontal lobe is a large and complex brain region composed of several subregions that have different functions in cognitive and emotional processes. We included the frontal lobe as a large brain region in the current study, which may overlook the differences between its subregions and their contributions to the underlying mechanisms of anxiety disorders in dogs. Future study can used other atlas, such as from Johnson and colleagues^115^, which parcellated frontal lobe into several subregions.

## Conclusions

In summary, using diffusion tensor imaging and graph theoretical approach, we detected alterations in the structural white matter connectome of anxious compared to healthy dogs. We found that dogs diagnosed with anxiety behavior showed a sparse and low inefficient structural brain network compared to the healthy group, indicating a disrupted network organization of the structural connectome in anxious dogs. Moreover, we found that the posterior parts of the brain are particularly involved in the neuropathological mechanisms of anxiety diagnosed dogs, which might share the similar neuropathology with humans. Given that the abnormal nodal properties in the left cerebellum may be related to the expression of excessive anxiety behaviors, this underscores the importance of the cerebellum translationally in behavioral abnormalities.

## Supplementary Information

Supplementary Tables.

## Data Availability

The datasets generated and analyzed in the current study are only made available on request from the corresponding author under a formal data sharing agreement.
