# Supplementary material for: Structural connectome alterations in anxious dogs: a DTI-based study
Source: Sci Rep. 2023 Jun 19;13:9946. doi: 10.1038/s41598-023-37121-0 (PMC10279662; doi:10.1038/s41598-023-37121-0)
Supplement: Supplementary file 1 — Supplementary Tables. [file 41598_2023_37121_MOESM1_ESM.docx]

**Structural connectome alterations in anxious dogs: A DTI-based study**

Qinyuan Chen, Yangfeng Xu, Emma Christiaen, Guo-Rong Wu, Sara De Witte, Christian Vanhove, Jimmy Saunders, Kathelijne Peremans, Chris Baeken

**Supplementary information**

**Table S1** Demographic information of anxious dogs

| **Subject ID** | **Breed** | **Age (months)** | **Gender** |
| --- | --- | --- | --- |
| 1 | Jack Russell terrier | 64 | FC |
| 2 | Belgian shepherd | 66 | MC |
| 3 | Galgo Espanol | 71 | FC |
| 4 | White Swiss Shepherd | 64 | FC |
| 5 | Akita Inu | 111 | FC |
| 6 | Labrador retriever | 90 | MC |
| 7 | Spanish water dog | 41 | MC |
| 8 | Galgo Espanol | 86 | MC |
| 9 | Belgian shepherd | 55 | FC |
| 10 | Border collie | 98 | MC |
| 11 | Jack Russell terrier | 151 | MC |

Abbreviations: FC, Castrated female; MC, Castrated male.

**Table S2** C-BARQ scores for the anxious dogs

| **Subject No** | **C-BARQ scores** | | | | | | | | | | | | |
| --- | --- | --- | --- | --- | --- | --- | --- | --- | --- | --- | --- | --- | --- |
|  | S1 | S2 | S3 | S4 | S5 | S6 | S7 | S8 | S9 | S10 | S11 | S12 | S13 |
| 1 | 1.5 | 0.8 | 0 | 2.63 | 0.5 | 3.5 | 1.5 | 1.83 | 0 | 0.5 | 2 | 1.67 | 2 |
| 2 | 2.25 | 0 | 0.63 | 2.88 | 1 | 3.5 | 0 | 0.17 | 0 | 0.75 | 2.83 | 0.17 | 2 |
| 3 | 1.75 | 0 | 0.38 | 0 | 2.5 | 1.75 | 4 | 3.83 | 0.13 | 0.25 | 1.17 | 3 | 1 |
| 4 | 3.5 | 3.2 | 0 | 2.5 | 0 | 2.5 | 0.75 | 0.67 | 0.88 | 0.5 | 1.67 | 3.17 | 2 |
| 5 | 2.88 | 1 | 0.25 | 1.25 | N/A | 2.25 | 1.5 | 0 | 0 | 1.5 | 1 | 2 | 0 |
| 6 | 1.5 | 0 | 0 | 0.75 | N/A | 1 | 3 | 2.83 | 0 | 0.5 | 2 | 0.33 | 0 |
| 7 | 3.63 | 2.3 | 0 | 1 | N/A | 1.5 | 0 | 0.5 | 0.5 | 1 | 2.33 | 2.67 | 0 |
| 8 | 0.13 | 0 | 0 | 4 | 0 | 1.25 | 3.75 | 3.83 | 0 | 0.5 | 1.67 | 0.17 | 1 |
| 9 | 3.75 | 0.7 | 0 | 0.13 | N/A | 1 | 1.5 | 2.67 | 0 | 1 | 2.33 | 3.67 | 4 |
| 10 | 2.13 | 0 | 0 | 1 | N/A | 2 | 0.5 | 2 | 0.13 | 1 | 2.67 | 1.67 | 1.5 |
| 11 | 1.38 | 0.3 | 0.25 | 2.5 | 0 | 0.25 | 1.25 | 0.67 | 1.88 | 3.25 | 2.67 | 3 | 2 |

Abbreviations: C-BARQ, Canine Behavioral Assessment & Research Questionnaire; S1, Trainability; S2, Stranger-directed aggression; S3, Owner-directed aggression; S4, Dog-directed aggression; S5, Familiar dog aggression; S6, Chasing; S7, Stranger-directed fear; S8, Nonsocial fear; S9, Separation-related problems; S10, Touch sensitivity; S11, Excitability; S12, Attachment/attention-seeking; S13, Energy.

**Table S3** Brain regions included in the parcellated atlas

| Index | Label | Index | Label |
| --- | --- | --- | --- |
| 1 | Temporal lobe L | 16 | Parietal lobe R |
| 2 | Parietal lobe L | 17 | Occipital lobe R |
| 3 | Occipital lobe L | 18 | Frontal lobe R |
| 4 | Frontal lobe L | 19 | Anterior cingulate gyrus R |
| 5 | Anterior cingulate gyrus L | 20 | Posterior cingulate gyrus R |
| 6 | Posterior cingulate gyrus L | 21 | Hippocampus R |
| 7 | Hippocampus L | 22 | Thalamus R |
| 8 | Thalamus L | 23 | Caudate nucleus R |
| 9 | Caudate nucleus L | 24 | Piriform lobe R |
| 10 | Piriform lobe L | 25 | Insular cortex R |
| 11 | Insular cortex L | 26 | Amygdala R |
| 12 | Amygdala L | 27 | Cerebellar hemisphere R |
| 13 | Cerebellar hemisphere L | 28 | Vermis R |
| 14 | Vermis L | 29 | Mesencephalon |
| 15 | Temporal lobe R | 30 | Diencephalon |

Abbreviations: L (R), left (right) hemisphere

**Table S4** Mathematical definitions and descriptions of topological parameters for a given network G

|  | **Parameters** | **Definitions** | **Descriptions** |
| --- | --- | --- | --- |
| **Global parameters** | Clustering coefficient | $C_{p}(G)=\frac{2}{k_{i}(k_{i}-1)}\sum_{j,k} {(\bar{w}_{ij}\bar{w}_{jk}\bar{w}_{ki})}^{1/3}$ | *Ki* is the degree of node *i*. The clustering coefficient *(Cp)* measures the fraction of a node's neighbors that are connected to each other and thus provides information about the connectedness of a node's neighbors. |
|  | Characteristic path length | $L_{p}(G)=\frac{1}{N(N-1)}\sum_{i=1}^{N} \sum_{j=1,i\neq j}^{N} L_{ij}$ | *Lij* is the shortest path length between node *i* and node *j* using a "harmonic mean" geodesic distance between all pairs of nodes. *Lp (G)* quantifies the ability of parallel information propagation or global efficiency of a network. |
|  | Global efficiency | $E_{glob}(G)=\frac{1}{N(N-1)}\sum_{i=1}^{N} \sum_{j=1,i\neq j}^{N} \frac{1}{L_{ij}}$ | *1/Lij* represents the reciprocal of the "harmonic mean" of shortest path length. Global efficiency *(Eglob)* is a measure of a network's capacity for parallel information transfer between nodes via multiple series of edges. |
|  | Local efficiency | $E_{loc}(G)=\frac{1}{N}\sum_{i=1}^{N} E_{glob}(G_{i})$ | *Eglob (Gi)* is the global efficiency of the subgraph *Gi* of the node *i*. The local efficiency reveals the degree of fault tolerance of the network, suggesting how efficient the communication is among the first neighbors of the node *i* when it is removed. |
|  | Small worldness | $\sigma= {C_{p}^{real}/C_{p}^{rand} \atop L_{p}^{real}/L_{p}^{rand}}$ | $C_{p}^{real}$: clustering coefficient of the real network. $C_{p}^{rand}$: mean clustering coefficient of 100 matched random networks that have the same number of nodes and edges as well as the degree distribution as the real network. $L_{p}^{real}$ is the characteristic path length of the real network, and $L_{p}^{rand}$ is the mean characteristic path length of 100 matched random networks. |
| **Nodal parameters** | Nodal strength | $K_{nod}\left( i \right)=\sum_{j=1,i\neq j}^{N} e_{ij}$ | *eij* is the edge for the node *i* with any connected nodes. The strength of a node is the sum of the network strengths across all the nodes over the whole network. |
|  | Nodal efficiency | $E_{nod}\left( i \right)=\frac{1}{N-1}\sum_{j=1,i\neq j}^{N} \frac{1}{L_{ij}}$ | *Lij* is the characteristic path length between nodes *i* and *j* in the network. *Enodal (i)* measures the average shortest path length between a given node *i* and all the other nodes in the network. |
|  | Betweenness centrality | $BC\left( i \right)=\sum_{k\neq i\neq j} \frac{\sigma_{jk}(i)}{\sigma_{jk}}$ | *δjk* is the number of shortest path lengths between node *j* and node *k*. *δjk (i)* is the number of shortest path lengths between node *j* and node *k* through node *i*. |
